# Extrapyramidal and anticholinergic reactions in an adolescent patient: a case for pharmacogenetic consideration

**DOI:** 10.11613/BM.2026.020902

**Published:** 2026-04-15

**Authors:** Iris Žunić Išasegi, Ivana Stefanović, Marta Okružnik Želalić, Livija Šimičević, Lana Ganoci, Maja Živković, Ivan Begovac, Nada Božina

**Affiliations:** 1 Department of Child and Adolescent Psychiatry, University Hospital Centre Zagreb, Zagreb, Croatia; 2 Division of Pharmacogenomics and Therapy Individualization, Department of Laboratory Diagnostics, University Hospital Centre Zagreb, Zagreb, Croatia; 3 School of Medicine, University of Zagreb, Zagreb, Croatia; 4 Department of Psychiatry and Psychological Medicine, University Hospital Centre Zagreb, Zagreb, Croatia

**Keywords:** borderline personality traits, child and adolescent psychiatry, extrapyramidal symptoms, anticholinergic syndrome, pharmacogenetics

## Abstract

Adolescent patients with borderline personality traits present a significant clinical challenge due to their polymorphic symptomatology occurring together with ongoing psychological and physiological development. While psychotherapy remains the first-line treatment, it is often combined with different psychopharmacological medications. This case report presents a 17.5-year-old female adolescent who developed extrapyramidal symptoms (EPS) following the addition of fluphenazine to her therapy, which had been modified due to deteriorating psychiatric symptoms. After the resolution of the EPS, the patient developed anticholinergic syndrome within a few days. Pharmacogenetic testing showed genetic variations associated with modified drug and dopamine metabolism, which may have contributed to her increased predisposition to drug adverse effects. This case highlights the complexity of psychopharmacological management in adolescents with multidimensional symptomatology and emphasizes the potential value of integrating pharmacogenetic data to guide optimal treatment strategies.

## Graphical abstract

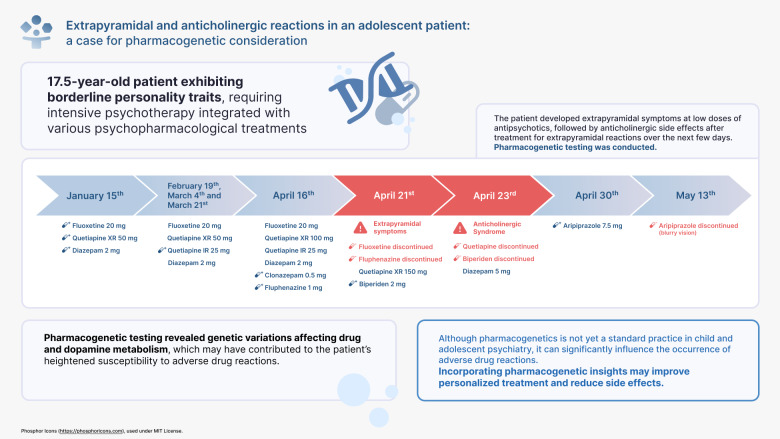

## Highlights

• The more polymorphic the symptoms, the more complex the treatment of adolescent patients

• An adolescent patient developed extrapyramidal symptoms after fluphenazine

• Patient developed anticholinergic syndrome after biperiden, following extrapyramidal symptoms from fluphenazine

• Pharmacogenetic testing showed CYP2C9 poor and CYP2D6 normal–intermediate metabolism

• Pharmacogenetic findings reveal patient characteristics, supporting genotype-guided therapy.

## Introduction

Treatment of patients with borderline personality disorder (BPD) traits in adolescence represents a great challenge because of their polymorphous symptomatology. Although psychotherapy remains the first line of treatment, an additional psychopharmacological approach, unfortunately often off label, is also required. In adolescents, off-label use of medications - prescribing drugs outside approved indications, age ranges, or dosages - is often necessary and considered ethically appropriate when guided by evidence, careful risk-benefit assessment, proper monitoring, and informed parental involvement. The greater the polymorphic nature of symptomatology, the more complex the treatment approach is required in adolescents. In cases of polymorphic symptomatology, priority is given to addressing the most severe and dangerous symptoms first. For example, in BPD traits, these include suicidality, poor self-image, and related manifestations. It is also common in clinical practice to implement a combination of therapeutic approaches and to adjust or change pharmacological treatments as needed. Many physiological changes take place during childhood and adolescence, which may affect the pharmacokinetics of psychotropic drugs (*1*-*4*). Consequently, adolescent psychopharmacotherapy warrants caution during treatment initiation and adjustment. Pharmacogenetic testing facilitates personalized and efficacious treatment strategies by elucidating inter-individual variability in drug effects attributable to genetic differences, thereby mitigating empirical prescribing, diminishing adverse effects, expediting therapeutic response, and enhancing the likelihood of remission.

The patient’s parents provided written informed consent during her treatment for the potential use and publication of clinically relevant data. This was done in accordance with the ethical standards of the institutional research committee and with the 1964 Helsinki Declaration and its later amendments, ensuring full protection of the patient’s identity and the confidentiality of all related data.

## Case description

This is a case report about 17.5-year-old girl with a history of psychiatric treatment since she was 13.5 years old. The patient presented with restrictive anorexia nervosa, which later expanded to polymorphous symptomatology. It included non-suicidal self-harm behavior often accompanied with high suicidal risk, gender dysphoria elements, bulimic behavior, and dissociative phenomena. Furthermore, she suffered from affective instability, unstable interpersonal relationships, chronic feelings of emptiness, and an intense fear of abandonment.

The clinical presentation of our patient required intensive psychotherapy sessions combined with psychopharmacological treatment. Over the course of four years, she experienced multiple inpatient stays, received care at a day hospital and recurrent outpatient treatment approximately every two weeks. Standard examinations were often performed, including monitoring the dynamics of blood tests, electrocardiograms, electroencephalograms, brain magnetic resonance imaging, and psychological testing. She was also continuously followed in pediatric care due to the potential somatic consequences of eating disorder symptoms.

**Figure 1:**
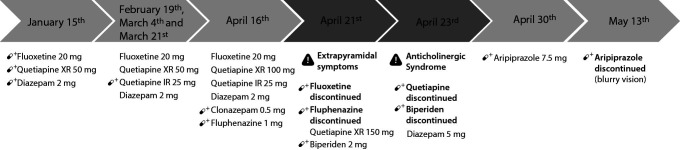
Timeline of administered medications and associated adverse drug reactions. DD - daily dose. (Icons from Phosphor Icons, licensed under the MIT License).

From January, the therapy included fluoxetine, quetiapine, and diazepam. The timeline of therapy changes, including daily doses, along with the occurrence of side effects, is presented in Figure 1. During an outpatient treatment session in April 2024, when she was 17.5 years old, she admitted to having experienced paranoid ideations for a long time, which caused her significant distress. At that time, her pharmacotherapy regimen included fluoxetine at daily dose (DD) of 20 mg, quetiapine XR (extended release) 50 mg, quetiapine IR (immediate release) 25 mg (administered at noon), clonazepam as needed at 0.5 mg. with a maximum dose of three tablets *per* day. Due to the observed anxious-related paranoid ideation and the evident progressive deterioration in her clinical presentation, mostly regarding BPD traits, pharmacotherapy was revised. The revised therapy included a gradual discontinuation of fluoxetine (20 mg administered every other day for 10 days, followed by complete cessation), an increase of quetiapine XR dose to 100 mg daily, quetiapine IR 25 mg on an as-needed basis (up to 2-3 times *per* day) and the introduction of fluphenazine in DD of 1mg. The clonazepam dosage remained unchanged (Figure 1).

Five days after the introduction of the new pharmacotherapy regimen, the patient and her mother contacted us by telephone, reporting symptoms indicative of acute dystonia. The patient was immediately invited and examined in our outpatient clinic the same day. She described muscle rigidity in the back, neck, and the thighs, with intermittent blurred vision, which had begun the previous day. Her legal guardian confirmed regular intake of the prescribed therapy and reported that she had difficulty walking with occasional backward movements of the neck. Our patient skipped her morning dose that day and her symptoms gradually improved by the time she arrived at our department. Physical examination revealed normal vital signs, with no symptoms of significant muscle rigidity or laryngeal spasm/dysphonia.

At the examination, a 2 mg dose of biperiden was administered, after which the patient reported subjective relief from muscle rigidity. Her pharmacotherapy was promptly revised: fluphenazine and fluoxetine were immediately discontinued, biperiden was initiated at 2 mg once daily for the following two days, the quetiapine XR dose was increased to 150 mg daily, and clonazepam was maintained at the current dose.

Two days later, patients mother contacted us again, and we recommended a pediatric evaluation through the emergency department because our patient was experiencing the following symptoms: headache, nausea without vomiting, alternating constipation and diarrhea, an intermittent feeling of suffocation with blurred vision when looking down and straight ahead, mydriasis, diplopia, difficulty urination. At the emergency department, the patient was examined by an ophthalmologist due to suspected narrow-angle glaucoma. The patient’s vital signs were within normal limits. Supportive somatic treatment was initiated, including the discontinuation of the current psychopharmacotherapy and the introduction of diazepam instead. The patient continued psychiatric treatment and was admitted to our department five days after the pediatric evaluation for pharmacological modification under inpatient conditions. Aripiprazole was gradually introduced during the inpatient stay, reaching daily dose of 7.5 mg, at which point the patient reported blurry vision. A follow-up ophthalmological examination suggested possible side effects of aripiprazole, after which aripiprazole was excluded from therapy. The patient was maintained on diazepam monotherapy. Considering the patient’s adverse drug reactions, a comprehensive pharmacogenetic panel was performed, targeting common functional polymorphisms in genes encoding drug-metabolizing enzymes and transporters. The analyzed pharmacogenes included CYP1A2, CYP2B6, CYP2C9, CYP2C19, CYP2D6, CYP3A4, CYP3A5, NAT2, ABCB1 (MDR1), ABCG2, as well as variants in the serotonin transporter gene promoter region (5-*HTTLPR*), catechol-O-methyltransferase (*COMT*), dopamine receptor D2 (*DRD2*), and the dopamine transporter variable number tandem repeat (*DAT1* VNTR). Pharmacogenetic testing was performed using a targeted panel, including TaqMan DME and SNP assays on a 7500 Real-Time polymerase chain reaction (PCR) System (Applied Biosystems, Thermo Fisher Scientific, Waltham, USA), while PCR and PCR- restriction fragment length polymorphism (RFLP) methods were used for the analysis of 5-HTTLPR, DRD2, and DAT1 VNTR. Genomic DNA was isolated from 3 mL of EDTA-anticoagulated peripheral blood using the QIAcube automated system (QIAGEN, Hilden, Germany).

## Pharmacogenetics findings

The genotyping results indicated the presence of genetic variations associated with altered drug metabolism as well as dopamine signaling. All pharmacogenetic findings are summarized in Table 1, presenting the patient’s pharmacogenetic profile and including polymorphisms of cytochrome P450 enzymes, transporter genes, and neurotransmitter-related genes, relevant for psychopharmacological treatment. Genotype-derived phenotypes are shown alongside known substrates or inhibitors of the listed enzymes or transporters. According to pharmacogenetic findings, both drug-metabolism and dopamine-related genetic factors, played a role in the patient’s response to treatment.

**Table 1:** Pharmacogenetic profile of the patient and related pharmacotherapy

**Gene/allele**	**Genotype**	**Phenotype**	**Drug substrate**	**Drug inhibitor**
CYP1A2*1F (-163C>A, rs762551)	*1F/*1F	Normal/Rapid metaboliser	Fluoxetine	Fluoxetine
*CYP2B6* *4,*6, *9	*1/*1	Normal metaboliser	Fluoxetine Diazepam	
*CYP2C9* *2,*3	*2/*2	Poor metaboliser	Fluoxetine Diazepam	Fluoxetine
*CYP2C19* *2, *17	*1/*1	Normal metaboliser	Fluoxetine Quetiapine Diazepam	Fluoxetine Diazepam
*CYP2D6* xN, *3, *4, *5, *6, *9, *10, *41	*1/*41	Normal/Intermediate metaboliser	Fluoxetine Quetiapine Fluphenazine Biperiden Aripiprazole	Fluoxetine Fluphenazine
*CYP3A4* *1B, *22	*1/*1	Normal metaboliser	Fluoxetine Quetiapine Clonazepam Diazepam Aripiprazole	Fluoxetine Diazepam
*CYP3A5* *3	*1/*3	Intermediate metaboliser	Fluoxetine Quetiapine Diazepam Aripiprazole	
*NAT2* *4, *5, *6	*5/*5	Poor metaboliser	Clonazepam	
*ABCB1* c.3435C>T	C/C	Normal function	Quetiapine Diazepam	Fluoxetine Fluphenazine Aripiprazole
*ABCG2* c.421C>A	C/**A**	Decreased function	Quetiapine	
*SLCO1B1* *5 (c.521T>C)	*1/*1	Normal function		
5-*HTTLPR* (L_A_, L_G_, S_A_)	L_A_/L_A_	High expression		
*COMT* c.472G>A (p.Vall58Met, rs4680)	G/G	Higher enzymatic activity		
*DAT1 VNTR* (6-12)	**9**/10	Intermediate expression		
*DRD2* (Taq1A, A1, A2)	A2/A2	Normal expression		
Variant alleles are bolded. *5-HTTLPR* - Serotonin transporter gene promoter region. *ABCB1* - ATP-binding cassette subfamily B member 1. *ABCG2* - ATP-binding cassette subfamily G member 2. *COMT* - Catechol-O-methyltransferase gene. *DRD2* - Dopamine receptor D2 gene. *DAT1 VNTR* - Dopamine transporter gene variable number tandem repeat. *NAT2* - N-acetyltransferase 2. *SLCO1B1* - Solute carrier organic anion transporter family member 1B1.				

## Discussion

This case report describes an adolescent patient with polymorphous psychic symptoms treated with low doses of antipsychotics but who at first developed EPS, and after biperiden treatment in combination with quetiapine developed elements of anticholinergic syndrome.

Extrapyramidal symptoms are common adverse effects of antipsychotics, and the development of acute EPSs could depend on the activity of dopaminergic system and its gene variants (*5*).

Our patient’s pharmacogenetic profile may clarify factors that contributed to the development of EPS following the introduction of fluphenazine to fluoxetine, quetiapine, and clonazepam treatment. The patient is a homozygous for the CYP2C9*2 allele and is predicted to be CYP2C9 poor metabolizer and heterozygous for the CYP2D6*41 allele, indicating CYP2D6 normal-to-intermediate metabolizer. Specifically, fluphenazine is metabolized by CYP2D6; moreover, fluoxetine is a potent inhibitor of both CYP2C9 and CYP2D6 (*6*-*9*). Moreover, the presence of the CYP2D6*41 allele with reduced enzymatic function, and concurrent fluoxetine therapy may have caused *phenoconversion*, a functional inhibition of CYP2D6 activity, actually changing the patients’ metabolizer status closer to poor metabolizer phenotype. This functional reduction in CYP2D6 activity may have secondarily impaired fluphenazine clearance, increasing the risk of adverse drug reactions (*10*-*12*).

This combination of genetic predisposition and drug-drug interaction plausibly reduced the metabolism of fluphenazine, resulting in elevated fluphenazine plasma concentrations increasing the risk of dopaminergic blockade and EPS development (*13*, *14*).

In addition to the pharmacokinetics factors described above, genetic variability may further modify the dopaminergic pharmacodynamic profile, contributing to dopaminergic hypoactivity. Predicted higher COMT activity (*COMT* c.472GG genotype) may lead to more rapid dopamine clearance in the prefrontal cortex, potentially lowering baseline dopaminergic tone and therefore increasing predisposition to EPS development (*15*). Furthermore, intermediate DAT1 expression (linked with *DAT1* 9/10 repeat) may enhance dopamine reuptake and thereby diminish dopamine availability at postsynaptic receptors (*16*, *17*). Finally, normal D2 receptor density (*DRD2 Taq1A* A2/A2 genotype) may not stimulate compensatory upregulation of receptor and may consequently further reduce dopaminergic signaling together with dopamine-blocking agents (*18*). These pharmacogenetic findings highlight both pharmacokinetic and pharmacodynamic characteristics of the patient and support the implementation of genotype-guided psychopharmacological therapy. In this case, future treatment should avoid potent CYP2C9/CYP2D6 inhibitors and antipsychotics with high affinity for the D2 receptor.

The underwent anticholinergic syndrome was anticipated, considering the pharmacological profiles of the administered therapy, quetiapine and biperiden, followed by aripiprazole. Quetiapine is primarily metabolized by the CYP3A4 enzyme and is also a substrate of the ABCG2 transporter. Patient’s decreased transport function of ABCG2 may increase quetiapine concentrations, potentially exacerbating anticholinergic side effects (*19*, *20*).

In this patient, the occurrence of blurred vision during the initiation of aripiprazole therapy aligns with previously reported cases in the literature, although the underlying mechanisms remain incompletely understood. Potential explanations include acute transient myopia secondary to ciliary body and choroidal effusion and swelling, anterior displacement of the ciliary processes, narrowing of the ciliary sulcus, anterior movement of the iris and lens, or direct drug penetration into the lens resulting in osmotic swelling (*21*). Given that aripiprazole is metabolized primarily *via* CYP2D6, and the patient is a normal to intermediate CYP2D6 metabolizer, these adverse effects may also be related to metabolic variability. This further highlights the importance of pharmacogenetic testing in cases of such side effects, as it allows for a more precise assessment of potential correlations and, possibly, causal relationships. BPD traits in adolescence are still topics of discussion in child and adolescent psychiatry, particularly considering that adolescence *per se* is a turbulent and emotionally challenging period. Nevertheless, the stability and reliability of symptoms and features (such as identity disturbance, affective instability, relationship difficulties, impulsivity) are high, and these traits interfere with normal developmental processes in adolescence. Hence, if neglected or unrecognized, they can evolve into chronic patterns with poor psychosocial outcomes in adulthood. Therefore, timely intervention is crucial for adolescents coping with these difficulties (*22*-*24*). Although psychotherapy remains the first-line treatment, medications are commonly prescribed for patients with BPD. Negative attitudes toward both psychotherapy and drug treatment are common among patients/parents, especially considering that clinical entity, which makes it even more important to select medications that relieve psychological difficulties without causing side-effects (*25*, *26*).

Although pharmacogenetics is not yet part of standard practice in child and adolescent psychiatry, it can considerably affect the adverse drug reactions. While pharmacogenetic guidelines for prescribing antidepressants and antipsychotics in the pediatric population are missing, it is hypothetically possible to implement Clinical Pharmacogenetics Implementation Consortium (CPIC) and Dutch Pharmacogenetics Working Group (DPWG) guidelines in pediatric psychiatry clinical practice to enhance therapeutic result and decrease adverse reactions risk, ultimately leading to better treatment outcomes (*11*, *27*-*29*). This clinical case illustrates how both drug-metabolism and dopamine-related genetic factors had a significant role in the patient’s response to treatment, highlighting the potential benefits of genotype-guided therapy in everyday clinical practice.

Our study has some limitations. The interpretation of the reported adverse drug reactions and pharmacogenetic findings is limited by the lack of drug blood concentration data. Therapeutic drug monitoring was not performed because the adverse effects appeared very soon after treatment initiation, at a stage when such measurements would have been unlikely to add meaningful clinical insight.

## Data Availability

No data was generated during this study, so data sharing statement is not applicable to this article.
